# Antimalarial drug use in general populations of tropical Africa

**DOI:** 10.1186/1475-2875-7-124

**Published:** 2008-07-08

**Authors:** Florence Gardella, Serge Assi, Fabrice Simon, Hervé Bogreau, Teunis Eggelte, Fatou Ba, Vincent Foumane, Marie-Claire Henry, Pélagie Traore Kientega, Léonardo Basco, Jean-François Trape, Richard Lalou, Maryse Martelloni, Marc Desbordes, Meïli Baragatti, Sébastien Briolant, Lionel Almeras, Bruno Pradines, Thierry Fusai, Christophe Rogier

**Affiliations:** 1 Unité de Recherche en Biologie et Epidémiologie Parasitaires (URBEP), IMTSSA, Parc du Pharo – B.P. 46, 13998, Marseille-Armées, France; 2 Unité de recherche sur les Maladies Infectieuses et Tropicales Emergentes (URMITE) – UMR 6236, Faculté de Médecine, 27 Bv Jean Moulin, 13385, Marseille, Cedex 05, France; 3 Institut Pierre Richet/Institut National de Santé Publique, BP V 47, Abidjan, Cote d'Ivoire; 4 Hôpital Laveran, BP 50, 13998, Marseille-Armées, France; 5 Dept. Infectious Diseases, AIDS and Tropical Medicine, Dep Clinical Pharmacology Meibergdreeef 15, 1105 AX, Amsterdam, The Netherlands; 6 UR 077 Paludologie Afrotropicale, BP1386, IRD, Dakar, Sénégal; 7 UR 077 Paludologie Afro-Tropicale, OCEAC, BP 288, Yaoundé, Cameroun; 8 Centre Muraz, BP 360, Bobo-Dioulasso, Burkina-Faso; 9 LPED, Université de provence, case 10, 3, place Victor-Hugo, 13331, Marseille, cedex 3, France; 10 Unité de Recherche en Pharmacologie et Physiopathologie Parasitaires, IMTSSA, Parc du Pharo – B.P. 46, 13998, Marseille-Armées, France

## Abstract

**Background:**

The burden of *Plasmodium falciparum *malaria has worsened because of the emergence of chloroquine resistance. Antimalarial drug use and drug pressure are critical factors contributing to the selection and spread of resistance. The present study explores the geographical, socio-economic and behavioural factors associated with the use of antimalarial drugs in Africa.

**Methods:**

The presence of chloroquine (CQ), pyrimethamine (PYR) and other antimalarial drugs has been evaluated by immuno-capture and high-performance liquid chromatography in the urine samples of 3,052 children (2–9 y), randomly drawn in 2003 from the general populations at 30 sites in Senegal (10), Burkina-Faso (10) and Cameroon (10). Questionnaires have been administered to the parents of sampled children and to a random sample of households in each site. The presence of CQ in urine was analysed as dependent variable according to individual and site characteristics using a random – effect logistic regression model to take into account the interdependency of observations made within the same site.

**Results:**

According to the sites, the prevalence rates of CQ and PYR ranged from 9% to 91% and from 0% to 21%, respectively. In multivariate analysis, the presence of CQ in urine was significantly associated with a history of fever during the three days preceding urine sampling (OR = 1.22, p = 0.043), socio-economic level of the population of the sites (OR = 2.74, p = 0.029), age (2–5 y = reference level; 6–9 y OR = 0.76, p = 0.002), prevalence of anti-circumsporozoite protein (CSP) antibodies (low prevalence: reference level; intermediate level OR = 2.47, p = 0.023), proportion of inhabitants who lived in another site one year before (OR = 2.53, p = 0.003), and duration to reach the nearest tarmacked road (duration less than one hour = reference level, duration equal to or more than one hour OR = 0.49, p = 0.019).

**Conclusion:**

Antimalarial drug pressure varied considerably from one site to another. It was significantly higher in areas with intermediate malaria transmission level and in the most accessible sites. Thus, *P. falciparum *strains arriving in cross-road sites or in areas with intermediate malaria transmission are exposed to higher drug pressure, which could favour the selection and the spread of drug resistance.

## Background

Malaria remains a major public health problem in Africa. Around 60% of 250–500 million clinical disease episodes and over 80% of 1.25 million deaths attributed each year to malaria occur in sub-Saharan Africa [1]. Several studies have described a two-fold increase in deaths due to malaria during the 1980s and 1990s because of the emergence of the chloroquine resistance [2-4]. However recent publications have documented a decline in malaria morbidity and mortality trends attributed to the increased access to artemisinin-based combination therapies and widespread use of insecticide-treated nets [5-7].

Drug pressure, intensity of malaria transmission and population movement favour the spread of antimalarial drug resistance [8-10]. Uncontrolled antimalarial drug use is a critical factor that contributes to the drug pressure. Exploring socio-cultural factors which influence antimalarial drug use has been recognized as a priority. Furthermore, since one of the objectives of Roll Back Malaria was to promote an equitable coverage and access of antimalarial drugs [11], the impact of environmental and behavioural factors on treatment use is important to be recognized. However, few studies have focused on this aspect of the epidemiology of drug-resistant malaria [12,13]. The distance to public health facilities, socio-economic level, age and parasite prevalence have been identified as key factors of drug use, but these factors have been described generally without taking into account each other simultaneously. Thus, the possible associations and interactions of these factors have never been explored. In order to evaluate the association between the use of antimalarial drug and geographical, socio-economic and behavioral factors, a multi center cross-sectional study was conducted in 2003 in 30 sites from three countries (Senegal, Burkina Faso and Cameroon), when CQ was still the first-line treatment of uncomplicated malaria. Although the sites are not formally representatives of the whole continent, they represent a wide panel of ecosystems and malaria endemicity conditions.

## Methods

### Study sites

**Figure 1:**
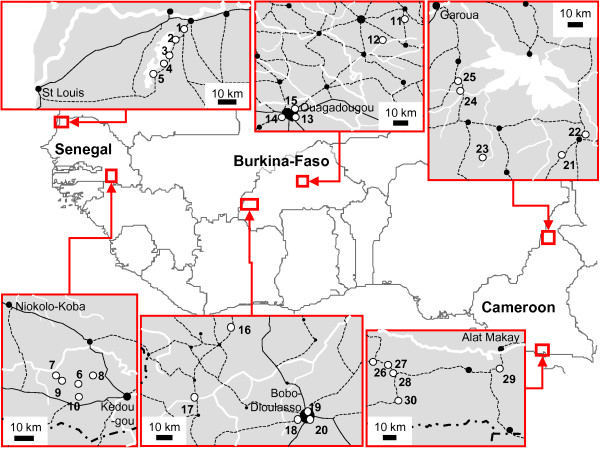
Map of the study areas in West and Central Africa. Study sites are indicated by open discs and their ID numbers on the maps of the study areas. Hydrographic networks are in white. Road networks are in black. Main localities are indicated by filled discs.

The study was conducted in two regions (in the north and the south of each country) in Senegal (sites #1 to 10), Burkina-Faso (sites #11 to 20) and Cameroon (sites #21 to 30) (Figure 1), between September 30 and December 17, 2003. In each area, this period corresponded to the end of the malaria transmission season or during the low transmission season. The rainy season (*i.e. *with an average of five or more rainy days per month in the nearest locality referred at ) lasts from August to September, from June to October, from May to September, from May to October and from May to October, in north Senegal, south Senegal, north Burkina-Faso, south Burkina-Faso and north Cameroon, respectively. In south Cameroon, there are two rainy seasons from March to June and from September to November. A list of different possible combinations of five sites (districts of towns or villages) was established for each region. The combinations were made to maximize the differences in environmental conditions suitable for malaria transmission, access to health structures and transport facilities between sites. A combination of five sites was randomly selected from the list of each region. In Burkina Faso, the combination of sites included three sites in an urban area and two sites in a rural area. In the regions of the other countries, only rural sites were included.

The informed consent of the parents of each child was obtained orally at the beginning of the study after a thorough explanation of its purpose. The study design received clearance from the Senegal (Dakar), Burkina Faso (Ouagadougou) and Cameroon (Yaoundé) National Ethics Committees.

### Site characteristics

In each site, 30 households were randomly selected from a numbered households list when it was available for the site or using a random-walk that was calibrated to be able to cover the whole of the surface of the site and started from its center.

The head of each household was interviewed on the number of individuals in the household, the number of rooms occupied, and the presence of the following facilities: running water, electricity, kitchen, refrigerator, living room, dining room, television, radio, video recorder, mobile, landline phone, vehicle, characteristics of the house (wall without anfractuosities, ground built with tiles or cement, windows that can be closed hermetically, roof built with a permanent structure). The number of these facilities that were present was calculated and used as an unweighted score from 0 to 16 reflecting the socio-economic level of each household.

A numbered list of all the inhabitants was drawn up for each household. Among them, up to three individuals were randomly chosen using a random numbers table. These individuals or their legal tutor were interviewed on their history of previous malaria attack, their personal characteristics (including age, last travel outside his/her village or town, length of residence in the site), their travels (number of nights spent in another village in the last 30 days) and their use of antimalarial drugs (including names of anti-malarial drugs commonly used, places where drugs are purchased, presence of stocks of medicines in the household). The sites were characterized by aggregating data collected from individuals or households (by calculating the median or proportion). Distances between sites and towns, sanitary structures and transport system were obtained from global positioning system coordinates. The duration of the corresponding travel were estimated by the averaged responses from key persons and heads of households.

### People

The consumption of antimalarial drug was estimated by testing the presence of chloroquine (CQ) and pyrimethamine (Pyr) parent compounds and metabolites in urine samples of 100 children between two and nine years of age randomly selected in each site, independently of their clinical status. Parental consent was obtained for each child. The test is based on an enzyme-linked immunosorbent assay (ELISA) blocking test, where immobilized antibody was first reacted with the test sample and then with a drug-peroxidase-enzyme conjugate and finally with the peroxidase-enzyme-substrate [14]. The sensitivity for the detection of CQ and Pyr were 20 ng/ml and 50 ng/ml, respectively. The specificity in negative controls was 100%. The presence of antimalarials in urine was also tested in a random sub-sample of urine from each country using an high-performance liquid chromatography (HPLC) technique. A numbered list of all the urines was drawn up for each country. Among them, up to hundred urine samples were randomly chosen using computer-generated random numbers and sent at -20°C to France and then kept at -80°C until analysis. Samples without sufficient volume of urines (*e.g. *that were spilt during the transport) were not processed. HPLC allows the detection of CQ (the sensitivity has been defined for each molecule), amodiaquine, quinine, mefloquine, halofantrine, proguanil, sulphadoxine, doxycycline and pyrimethamine, as described elsewhere [15,16].

Fingerprick capillary blood was obtained to prepare Giemsa-stained thick smears. Parasite density and the number of trophozoites against 100 leukocytes were calculated for each Plasmodium species. Blood spots were collected and dried on Whatman^® ^3 MM filter paper. IgG antibody against *P. falciparum *circumsporozoite protein (CSP) was measured in dried blood samples using elution and ELISA techniques described elsewhere [17,18]. Sites were classified according the prevalence of anti-CSP antibodies: low (less than 20%), intermediate (20 – < 40%) and high (≥ 40%).

For each child, information on site, age, sex, clinical status (fever during the last three days), consumption of anti-malarial drugs during the last seven days, travel during the last month was collected by questionnaires.

### Statistical methods

Data were entered using EpiDATA v3.0 [19] and checked for consistency before statistical analysis using R 2.5.0. Descriptive analysis was done to determine the level of use of CQ and PYR, of anti-CSP antibodies, malaria or parasite rate and other characteristics by site.

The presence of CQ in urine was analysed as a dependent variable according to individual and site characteristics using a random-effect logistic regression model to take into account the interdependency of observations made within the same site.

A bivariate analysis was first performed by entering each independent variable in the logistic model. Variables were retained for the multivariate analysis when their effects had a p-value lower than 0.25. A multivariate analysis was done in two steps. First, an empty regression model was developed to evaluate the between-sites random variation. This was followed by the selection of children and site characteristics in the bivariate analysis and added to the model. Then a multivariate analysis was performed by a backward step-by-step procedure. The independent variables and their interactions were retained in the model if their effects were significant (likelihood ratio statistic, p < 0.05). The adequacy of the final model was estimated by the area under the receiver operating characteristic (ROC) curve and by looking at the adequacy between observed and predicted probabilities of detecting CQ in children's urine samples in each site.

## Results

### Site description

In the 30 sites, 3,231 children between two and nine years of age and 3,097 individuals from 1,109 households were randomly selected (Table 1). The prevalence of *P. falciparum *trophozoites was significantly (p < 0.05) different between sites. It varied from 16.2% (site 2) to 96.1% (site 18) (Table 2), with a median of 72%. It was significantly higher in the south than in the north of Senegal (72% versus 25%, p < 10^-3^), Burkina Faso (79% versus 64%, p < 10^-3^), and Cameroon (78% versus 61%, p < 10^-3^), and also higher in rural than in urban areas of Burkina Faso (84% versus 64%, p < 10^-3^). It was higher among children older than five years than among children below five years of age (65% versus 59%, p = 0.002).

**Table 1:** Study sites. ID number, country, region, type of area and geographical coordinates.

**Site's name**	**ID**	**Country**	**Region**	**type of ** **area**	**Latitude** degree	**Longitude ** degree
THIAGO	1	Senegal	North	rural	16.4	-15.72
TEMEYE SALANE	2	Senegal	North	rural	16.33	-15.77
SANINTE	3	Senegal	North	rural	16.23	-15.8
NDIAKHAYE	4	Senegal	North	rural	16.18	-15.82
MALLA	5	Senegal	North	rural	16.12	-15.87
TIABEDJI	6	Senegal	South	rural	12.63	-12.4
SAMAL	7	Senegal	South	rural	12.67	-12.5
THIOBO BANTATA	8	Senegal	South	rural	12.67	-12.33
ASSONI	9	Senegal	South	rural	12.65	-12.49
LANDE RUNDE. LANDE BAITIL	10	Senegal	South	rural	12.55	-12.40
TIPTENGA	11	Burkina Faso	North	rural	13.09	-0.81
FATIN	12	Burkina Faso	North	rural	12.93	-0.95
OUAGADOUGOU S29–30	13	Burkina Faso	North	urban	12.35	-1.47
OUAGADOUGOU PISSY S17	14	Burkina Faso	North	urban	12.34	-1.56
OUAGADOUGOU NIOKO II S26	15	Burkina Faso	North	urban	12.42	-1.47
NIENA	16	Burkina Faso	South	rural	11.72	-4.72
TENASSO	17	Burkina Faso	South	rural	11.28	-4.93
BOBO-DIOULASSO SAMAGAN	18	Burkina Faso	South	urban	11.13	-4.35
BOBO-DIOULASSO DOGONA	19	Burkina Faso	South	urban	11.2	-4.28
BOBO-DIOULASSO KUINIMA	20	Burkina Faso	South	urban	11.15	-4.28
YOUKOUT	21	Cameroon	North	rural	8.29	14.09
TCHOLLIRE II	22	Cameroon	North	rural	8.45	14.26
SAKDJE	23	Cameroon	North	rural	8.27	13.65
BOCKI	24	Cameroon	North	rural	8.75	13.53
KATE	25	Cameroon	North	rural	8.78	13.52
MELEN/NKOLAFENDEK	26	Cameroon	South	rural	2.77	12.52
MIATTA/DJOUZE	27	Cameroon	South	rural	2.73	12.63
ENDEGUE/ABDELON	28	Cameroon	South	rural	2.69	12.64
ZOEBEFAM/NKOLEMBOULA	29	Cameroon	South	rural	2.72	13.34
YEN/MEBAN II	30	Cameroon	South	rural	2.43	12.67

**Table 2:** Prevalence of *Plasmodium falciparum *trophozoites, anti-CSP antibodies and antimalarial drugs detected in children between two and nine years of age.

**ID Site**	**Nb of thick smears**	**Prevalence of trophozoites (all species)**	**Prevalence of *Plasmodium falciparum *trophozoites**	**Prevalence of anti-CSP antibodies**		**Detection of antimalarial drugs in children's urines**			
				
		Nb of thick smears + (%)	Nb of thick smears + (%)	Nb of serology	Nb of serology + (%)	CQ*		**PYR†**	
		
						CQ+‡	Prevalence (95% CI)	PYR+§	Prevalence (95% CI)
1	100	26 (26.0)	25 (25.0)	100	13 (13.0)	36	36.0 (26.6–46.2)	0	0.0 (0.0–3.6)
2	105	17 (16.2)	17 (16.2)	105	10 (9.5)	18	18.0 (11.0–26.9)	0	0.0 (0.0–3.6)
3	111	21 (18.9)	19 (17.1)	110	13 (11.8)	16	14.5 (8.5–22.5)	0	0.0 (0.0–3.3)
4	120	23 (19.2)	22 (18.3)	118	12 (10.2)	56	47.5 (38.2–56.9)	0	0.0 (0.0–3.1)
5	101	54 (53.5)	47 (46.5)	100	25 (25.0)	26	25.7 (17.6–35.4)	2	2.0 (0.2–7.0)
6	100	79 (79.0)	72 (72.0)	100	55 (55.0)	20	20.0 (12.7–29.2)	0	0.0 (0.0–3.6)
7	100	86 (86.0)	79 (79.0)	100	48 (48.0)	9	9.0 (4.2–16.4)	0	0.0 (0.0–3.6)
8	103	89 (86.4)	83 (80.6)	103	75 (72.8)	14	14.0 (7.9–22.4)	0	0.0 (0.0–3.6)
9	84	60 (71.4)	57 (67.9)	84	39 (46.4)	18	21.4 (13.2–31.7)	0	0.0 (0.0–4.3)
10	110	78 (70.9)	66 (60.0)	110	51 (46.4)	10	9.1 (4.4–16.1)	0	0.0 (0.0–3.3)
11	102	90 (88.2)	90 (88.2)	104	93 (89.4)	47	46.1 (36.2–56.2)	1	1.0 (0.0–5.3)
12	102	93 (91.2)	88 (86.3)	102	98 (96.1)	33	33.7 (24.4–43.9)	0	0.0 (0.0–3.7)
13	102	41 (40.2)	41 (40.2)	104	21 (20.2)	98	90.7 (83.6–95.5)	1	0.9 (0.0–5.1)
14	102	42 (41.2)	38 (37.3)	105	22 (21.0)	89	80.2(71.5–87.1)	0	0.0 (0.0–3.3)
15	100	69 (69.0)	68 (68.0)	101	24 (23.8)	60	61.9 (51.4–71.5)	0	0.0 (0.0–3.7)
16	117	100 (85.5)	99 (84.6)	115	82 (71.3)	18	16.7 (10.2–25.1)	0	0.0 (0.0–3.4)
17	102	79 (77.5)	78 (76.5)	102	88 (86.3)	18	20.0 (12.3–29.8)	1	1.1 (0.0–6.0)
18	102	98 (96.1)	98 (96.1)	105	93 (88.6)	18	18.4 (11.3–27.5)	0	0.0 (0.0–3.7)
19	101	88 (87.1)	88 (87.1)	104	78 (75.0)	31	31.3 (22.4–41.4)	0	0.0 (0.0–3.7)
20	104	55 (52.9)	53 (51.0)	104	30 (28.8)	66	68.8 (58.5–77.8)	1	1.0 (0.0–5.7)
21	101	65 (64.4)	55 (54.5)	99	23 (23.2)	49	51.0 (40.6–61.4)	20	20.8 (13.2–30.3)
22	103	83 (80.6)	80 (77.7)	66	25 (37.9)	89	89.0 (81.2–94.4)	0	0.0 (0.0–3.6)
23	101	79 (78.2)	72 (71.3	99	46 (46.5)	35	34.7 (25.5–44.8)	1	1.0 (0.0–5.4)
24	101	65 (64.4)	59 (58.4)	99	32 (32.3)	68	67.3 (57.3–76.3)	0	0.0 (0.0–3.6)
25	100	52 (52.0)	41 (41.0)	86	16 (18.6)	91	87.5 (79.6–93.2)	0	0.0 (0.0–3.5)
26	101	86 (85.1)	74 (73.3)	102	29 (28.4)	56	55.4 (45.2–65.3)	0	0.0 (0.0–3.6)
27	99	83 (83.8)	81 (81.8)	48	8 (16.7)	28	27.2 (18.9–36.8)	0	0.0 (0.0–3.5)
28	104	87 (83.7)	84 (80.8)	95	21 (22.1)	42	39.6 (30.3–49.6)	0	0.0 (0.0–3.4)
29	103	81 (78.6)	76 (73.8)	103	22 (21.4)	25	24.3 (16.4–33.7)	0	0.0 (0.0–3.5)
30	107	91 (85.0)	86 (80.4)	107	34 (31.8)	78	72.9 (63.4–81.0)	2	1.9 (0.2–6.6)
Total	3088	2060 (66.7)	1936 (62.7)	2980	1226 (41.1)	1262	41.3 (39.6–43.0)	29	1 (0.6–1.4)

Nb: number; %, percent, *CQ: Chloroquine, † PYR: Pyrimethamine, ‡ CQ+ = number of samples with chloroquine in urine, **§**PYR+: number of samples with pyrimethamine in urine, Prevalence: expressed in percent.

The prevalence of anti-CSP-antibodies varied from 9.5% (sites 2 and 4) to 96.1% (site 12) (Table 2) according to sites, with a median of 31%. It was significantly higher in the south than in the north in Senegal (54% versus 14%, p < 10^-3^) and in Burkina Faso (70% versus 49%, p < 10^-3^). In Cameroon, it was lower in the south (25% versus 32% p < 10^-3^). It was significantly higher in rural than in urban areas of Burkina Faso (86% versus 44%, p < 10^-3^) and higher among children aged more than five years old than among children aged less than five years (48% versus 32%, p < 10^-3^).

The time necessary to join the nearest tarmacked road varied from 0 to 8.5 hours with a median of one hour. The proportion of inhabitants who lived in another site one year before the survey varied from 0% to 25% with a median of 4.3%. The average index of the household socio-economic level varied from 0.9 to 8.7 with a median of 3. No systematic distribution of antimalarial drugs to the children had been organized in the sites during six previous months. The others sites characteristics (*i.e. *individual or household data aggregated by site) are presented in Additional Files 1 and 2.

### Chloroquine in urine

CQ was tested in urine samples by dipstick in 3,052 of 3,231 children, aged 2–9 years (no urine was available for 179 children, *i.e. *5% of the randomized children). Males represented 49.9% of the children for whom urine samples were available. The characteristics of the other children are detailed in Additional File 3. Among these 3,052 children, 41.4% had CQ in their urine (1262/3052). The prevalence of CQ in children urine samples varied from 9.0% (site 7) to 90.1% (site 13) with a median of 32.2%. The prevalence of CQ in urine was significantly different between countries (Senegal = 22%, Burkina Faso = 47% and Cameroon = 55%, p < 10^-3^), between regions within the same country (*i.e. *higher in the north than in the south in Senegal [29% versus 14%, p < 10^-3 ^], in Burkina Faso, [63% versus 31%, p < 10^-3 ^], and in Cameroon, [66% versus 44%, p < 10^-3 ^]) and between sites from the same region (Table 2). It was significantly higher in urban than in rural areas of Burkina Faso (59% versus 37%, p = 0.047).

The prevalence of CQ in urine samples was higher in sites with a moderate prevalence rate of anti-CSP antibodies (61%) than in sites with a low (39%, p <0.026) or high prevalence rate of anti-CSP antibodies (23%, p = 0.088).

**Figure 2:**
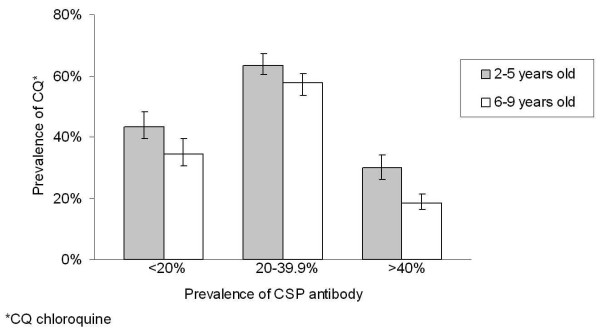
Prevalence rate (and 95% confidence interval) of chloroquine in urines of children between two and nine years of age according to their age and the prevalence rate of anti-CSP antibody among the children of the sites. *CQ: chloroquine.

The prevalence of CQ in children's urine was higher in those aged ≤ 5 years old than in children aged > 5 years old (49% versus 35%, p = 0.001). This difference was observed independently of the prevalence of anti-CSP antibodies in the sites (Figure 2).

The prevalence of CQ in urine was higher in children with a history of fever during the three days before urine sampling than in children with no history of fever (51% versus 38%, p = 0.032), and in children who had traveled out of the site during the month before urine sampling than children who did not leave the site (48% versus 41%, p = 0.048).

The prevalence of CQ in urine was higher in sites with more than 5% of inhabitants living in another site one year before urine sampling (49% versus 33%, p = 0.058), in sites with an average socio-economic level equal to 6 or higher (68% versus 36%, p = 0.002) and in sites in which the duration to join the nearest tarmacked road was less than one hour (48% versus 36%, p = 0.206). The other results of the bivariate analysis are presented in Tables 3 and 4 and in Additional File 4.

**Table 3:** Chloroquine prevalence in urines of children between two and nine years of age according to children characteristics.

**Variables**		N*	**CQ+†**	**Prevalence of** **presence of CQ‡ %**	**crude OR**	**95%CI**		**p**
**Sex**	Male	1524	618	41	1.00			
	Female	1528	644	42	1.07	0.90	1.26	0.458
**Age**	2–5 years old	1419	691	49	1.00			
	6–9 years old	1633	571	35	0.74	0.63	0.89	0.001
**Fever during the preceeding 3 days**	without	2252	852	38	1.00			
	with	800	410	51	1.24	1.02	1.50	0.032
**Antimalarial treatment during the preceding 7 days**	no	2652	995	38	1.00			
	yes	400	267	67	1.90	1.46	2.47	0.000
**Travel during the preceding 30 days**	no	2930	1203	41	1.00			
	yes	122	59	48	1.52	1.00	2.30	0.048
**Malaria infection**	no	974	466	48	1.00			
	yes	1939	718	37	0.60	0.49	0.74	0.000
**Asexual *Plasmodium falciparum infections***	no	1094	518	47	1.00			
	yes	1819	666	37	0.61	0.50	0.75	0.000

*N number of samples of urine, † CQ+ = number of samples with chloroquine in urines, ‡ CQ: Chloroquine.

Logistic regression model with random effect taking into account the interdependency of observations made within the same site.

**Table 4:** Chloroquine prevalence in urines of children between two and nine years of age according to site's characteristics.

**Site's characteristics**		N*	**CQ+†**	**Prevalence of CQ‡ %**	**crudeOR**	**95%CI**		**p**
Country	Senegal	1023	223	22	1.00			
	Burkina Faso	1007	478	47	3.69	1.49	9.16	0.005
	Cameroon	1022	561	55	5.54	2.23	13.72	0.000
Region	North	1547	811	52	1.00			
	South	1505	451	30	0.31	0.15	0.66	0.002
Type of area	rural	2443	900	37	1.00			
	urban	609	362	59	2.93	1.01	8.46	0.047
Prop. ‡ living in an other locality one year before the study	< 5%	1424	471	33	1.00			
	>= 5%	1628	791	49	2.29	0.97	5.40	0.058
Prop. ‡ living in an other site for more than 1 month during the preceding year	< 15%	1920	685	36	1.00			
	>= 15%	1132	577	51	2.24	0.92	5.45	0.077
Proportion of visitors among individuals present in the households the night before	< 2%	1918	635	33	1.00			
	>= 2%	1134	627	55	3.02	1.29	7.07	0.011
Prop. ‡ had a not damaged bed-net	< 30%	1826	868	48	1.00			
	>= 30%	1226	394	32	0.48	0.20	1.15	0.100
Prop. ‡ had access to stockpiles of antimalarial drugs at home	< 20%	1955	605	31	1.00			
	>= 20%	1097	657	60	4.05	1.86	8.81	0.000
Average number of individuals by household	< 7	834	501	60	1.00			
	7–9	1293	504	39	0.35	0.13	0.93	0.035
	>= 10	925	257	28	0.21	0.07	0.61	0.004
Socioeconomic level score in 2 classes	<6	2540	914	36	1.00			
	>= 6	512	349	68	4.73	1.74	12.87	0.002
Length of the travel to join the nearest sanitary structure	< 1 km	404	304	75	1.00			
	1–9.9 km	1034	425	41	0.20	0.06	0.67	0.010
	>= 10 km	1614	533	33	0.13	0.04	0.42	0.001
Length of the travel to join the pharmacy the most used	< 5 km	504	340	67	1.00			
	5–9.9 km	1034	425	41	0.29	0.09	0.95	0.041
	>= 10 km	1514	497	33	0.19	0.06	0.57	0.003
Duration of the route to join the nearest tarmacked road	< 1 hour	1336	640	48	1.00			
	>= 1 hour	1716	622	36	0.56	0.23	1.37	0.206
Prevalence rate of the anti-CSP antibodies among children between two and nine years of age	< 20%	635	245	39	1.00			
	20–39.9%	1227	746	61	2.81	1.13	6.97	0.026
	>= 40%	1190	271	23	0.44	0.17	1.12	0.088
Prevalence rate of *P. falciparum *trophozoites among children between two and nine years of age	< 25%	428	126	29	1.00			
	25–49%	526	351	67	6.62	1.67	26.23	0.007
	50–74%	989	407	41	1.76	0.52	5.92	0.359
	>= 75%	1109	378	34	1.27	0.38	4.21	0.693

*N number of samples of urine, † CQ+ = number of samples with chloroquine in urines, ‡ Prop.: proportion of inhabitants of the site who

Logistic regression model with random effect taking into account the interdependency of observations made within the same site.

There was no significant interaction between variables retained in the model. In multivariate analysis, the prevalence of CQ in urine was lower among children above five years of age (OR = 0.76, 95% CI = 0.64–0.90), and in sites in which the duration to join the nearest tarmacked road was one hour or more (OR = 0.49, 95% CI = 0.27–0.89). It was higher among children who declared a febrile episode during the three days preceding the urine sampling (OR = 1.22, 95% CI = 1.01–1.49), in sites with a high average socio-economical level (OR = 2.74, 95% CI = 1.11–6.78), in sites with more than 5% of inhabitants living in another site one year before urine sampling (OR = 2.53, 95% CI = 1.11–6.78) and in sites with a prevalence rate of anti-CSP antibodies among two to nine-year old children comprised between 20 and 39.9% (OR = 2.47, 95% CI = 1.13–5.41) (Table 5). The area under the ROC curve was 0.764. Additional File 5 shows the adequacy between observed and expected prevalence of CQ in urine according to the final model.

**Table 5:** Multivariate analysis of the presence of chloroquine in urines of children between two and nine years of age.

**Variables**	**N**	CQ+*	**Prevalence of CQ † %**	**Crude OR**	**95%CI**		**p‡**	**Adjusted OR**	**95%CI**		**p‡**
Age											
2–5 years old	1419	691	49	1.00				1.00			
6–9 years old	1633	571	35	0.74	0.09	0.63	0.001	0.76	0.64	0.90	0.002
Fever during the preceding 3 days											
without	2252	852	38	1.00				1.00			
with	800	410	51	1.24	1.02	1.50	0.032	1.22	1.01	1.49	0.043
Proportion of individuals who were living in an other locality one year before the study												
< 5%	1424	471	33	1.00				1.00			
>= 5%	1628	791	49	2.29	0.97	5.40	0.058	2.53	1.38	4.64	0.003
Score in 2 classes representing the households' average socioeconomic level											
< 6	2540	914	36	1.00				1.00			
>= 6	512	349	68	4.73	1.74	12.87	0.002	2.74	1.11	6.78	0.029
Prevalence rate of the anti-CSP antibodies												
< 20%	635	245	39	1.00				1.00			
20–39.9%	1227	746	61	2.82	1.11	7.16	0.0289	2.47	1.13	5.41	0.023
>= 40%	1190	271	23	0.44	0.17	1.12	0.0885	0.68	0.32	1.43	0.305
Duration of the route to join the nearest tarmacked road											
< 1 hour	1336	640	48	1.00				1.00			
>= 1 hour	1716	622	36	0.56	0.23	1.37	0.206	0.49	0.27	0.89	0.019

*CQ+ = number of samples with chloroquine in urines, † CQ: Chloroquine, ‡ p: p-value

Logistic regression model with random effect taking into account the interdependency of observations made within the same site.

### Pyrimethamine in urine

PYR was tested using the same dipstick as CQ. The prevalence of PYR in children's urine samples varied from 0% to 21% (site 21) with a median of 0%. It was 0.2%, 0.4% and 2.2% in Senegal, Burkina Faso and Cameroon, respectively. Because of the low prevalence rate of PYR in urine, no bivariate or multivariate analysis was done.

### Detection of antimalarials in urines using HPLC

HPLC measurement of CQ was performed for 280 urine samples (*i.e. *93, 98 and 89 children from Senegal, Burkina Faso and Cameroon, respectively). The prevalence of CQ detected by HPLC was 27%, 45% and 51% in Senegal, Burkina Faso and Cameroon, respectively. These prevalence rates were not significantly different from those estimated using dipsticks.

The prevalence of PYR detected by HPLC was 0%, 2% and 3% in Senegal, Burkina Faso and Cameroon, respectively. Amodiaquine was detected by HPLC in 6% (16/280) of the urine samples. Its prevalence rate was 2%, 8% and 7% in Senegal, Burkina Faso and Cameroon, respectively. Quinine was detected by HPLC in 1% (3/280) of the urines. Its prevalence rate was 0%, 1% and 2% in Senegal, Burkina Faso and Cameroon, respectively. Mefloquine, halofantrine, proguanil, sulphadoxine and doxycycline were not detected in any of the samples.

## Discussion

CQ was the first-line antimalarial drug used in 2003 in Senegal, Burkina Faso and Cameroon among children between two and nine years of age. Other studies had shown that CQ was the main antimalarial drug used in Africa [20-22]. For example, in the study of Talusina *et al *conducted in 1998 and 1999 in Uganda, the prevalence of CQ in urine obtained from children between one and nine years of age was 48% [10]. According to the sites, CQ was present in 9% to 90% (median 32%) of the urine samples collected in the present study. One of the significant findings of the study was the wide range of CQ prevalence observed from one site to another, including within the same region. Six factors associated with the heterogeneity of antimalarial drug use were identified.

### History of fever, age and socio-economic level

Three of these factors were expected. The first expected factor was the history of fever in days preceeding urine sampling. In case of fever, parents usually administer antimalarial drugs to their children as a presumptive treatment [22-24]. Second, the prevalence of CQ in urine was lower among children older than five years, most of whom have acquired antimalarial immunity during the first five years of permanent residence in an endemic area [25,26]. This association between age and CQ consumption was observed independently of the anti-CSP antibodies prevalence rate, *i.e. *the level of malaria transmission. The third expected factor was the average socio-economic level. High socio-economic level is associated with the ability to seek health service. In the study by Biritwun *et al *[27], conducted in Ghana, children from poorer community were less likely to take antimalarial treatment in a clinic or hospital as compared with children from a better-off community. These three factors are similar to those identified in earlier studies on the treatment given for fever [28,29].

### Population mobility

Three less expected factors associated with CQ consumption have been identified in the present study. First, the prevalence of CQ in children's urine was higher in sites where the proportion of inhabitants living in another site one year ago was higher. Thus, drug pressure was highest in sites where population migration was most frequent. Second, the prevalence of CQ intake was higher in sites where the duration to join the nearest tarmacked road was shorter. Thus, drug pressure was highest in most accessible sites. It is possible that accessibility by tarmacked road facilitates access to health services, independently of the socio-economic level. These two factors indicate that population mobility in relation to migration and site accessibility is positively associated with a more frequent antimalarial drug use. Two consequences may be expected: i) probability of resistant *P. falciparum *strain imported from another region or country is higher, ii) selective drug pressure on the Plasmodium population is higher. It could have facilitated the diffusion of chloroquino-resistance [8,30]. It is the first time that antimalarial drug use is shown to be associated with population mobility.

### Malaria transmission

The last factor associated with the presence of CQ in children's urine was the level of anti-CSP antibodies. The prevalence of anti-CSP antibodies was used as a proxy of the level of intensity of malaria transmission. This level is usually measured by determining the entomological inoculation rate. Parasite prevalence can be used as an alternative proxy [31], but in the present study the prevalence of anti-CSP antibodies was preferred because it is not modified by antimalarial treatment [32] or parasite resistance to drugs. CSP is actively expressed only during the sporozoite stage and is generally used as a proxy of the level of exposure to malaria [33]. In the present study, the prevalence of CQ in children's urine was higher in sites where the prevalence of anti-CSP antibodies was intermediate (between 20 and 39.9%). There are contrasting views on the role of transmission intensity of *P. falciparum *on drug consumption and spread of CQ resistance. In the study by Talisuna *et al*, conducted in seven sites in Uganda and involving 1,504 people aged 1–45 years, CQ use in all ages was inversely related to parasite prevalence [10]. The authors attributed this association with parasite prevalence, *i.e. *malaria endemicity, to the more rapid acquisition of antimalarial immunity in areas where the exposure to malaria infection is higher. A limitation of this study was the use of the parasite prevalence as the indicator for transmission intensity: this variable could be biased by drug use and drug resistance. In the present study, intermediate prevalence rate of anti-CSP antibodies, *i.e. *intermediate level of transmission, was significantly associated with higher consumption of CQ. It is consistent with the observation of Trape and Rogier who have reported that the cumulated incidence of clinical malaria was higher in intermediate endemic areas [4].

In terms of the spread of CQ resistance, there are three conflicting hypotheses on the role of malaria transmission [8,9]. The first hypothesis suggests that low transmission level increases the rate of spread of resistance because resistance gene combinations would be more stable and hence spread faster [34,35]. The second suggests that resistant parasites spread faster when transmission is high if intra-host dynamics exist: the increasing transmission intensity can increase the number of co-infecting clones, and antimalarial drug use would eradicate the drug susceptible clones and allow the survival of the resistant clones [35]. The third hypothesis suggests that the intensity of transmission intensity plays no role in the early stages of the evolution of parasite resistance [36]. All of these hypotheses do not take into consideration the effect of drug use. The present study shows that drug selection pressure was different between sites with different levels of transmission intensity. This observation should be taken into account for modeling the spread of drug resistance in relation to malaria transmission and acquisition of clinical immunity.

### Diversity of antimalarial drug use

Few drugs other than CQ were present in the urine samples analysed in the present study. The prevalence of pyrimethamine in urine ranged from 0 to 21% (median 0%), depending on the sites. This could at least partly explain the low level of antifolate resistance during the study. In the meta-analysis of Talusina *et al*, the median of the prevalence of sulphadoxine-pyrimethamine (SP) treatment failure in Africa between 1996 and 2002 varied from 0 to 35% [8]. The widespread adoption of intermittent preventive treatment using SP in pregnant women could lead to an increased prevalence of resistances to this drug in the next future. Since 2001 the World Health Organization recommends that treatment policies in all countries experiencing resistance of *P. falciparum *to conventional monotherapies should be combination therapies, preferably those containing artemisinin derivatives [37]. However, a change in national antimalarial treatment policies can take several years. In 2003, CQ was still the usual treatment in the three countries where the present study was conducted.

### Evaluating drug consumption

To assess the level of antimalarial drug consumption, two methods can be used: questionnaires and biological methods for detecting drugs in urine or blood. The assessment of antimalarial drug consumption by questionnaires is less reliable than biological methods because of misunderstanding of questions, failed memory, or deliberate attemps to provide false information [10,13,20]. In the present study, drug consumption was assessed by a CQ- and PYR-specific dipstick. The urine dipstick detects the presence of CQ and PYR and, by cross-reaction, also detects amodiaquine and proguanil. However, the standard method to detect and measure antimalarial drugs in urine and blood is high-performance liquid chromatography (HPLC). Since the latter is more expensive than urine dipstick, it was not used for all samples in this study. Proguanil was not found in urine by HPLC, and amodiaquine was present in only 6% of the samples. Therefore, the analysis of antimalarial consumption seems not biased by cross reactions.

## Conclusion

Antimalarial drug pressure considerably varied from one site to another, including within the same region, and was significantly higher in areas with intermediate malaria transmission level, *i.e. *where the level of acquired malaria immunity is intermediate, and in the most accessible sites. Therefore, incoming resistant *P falciparum *strains from other sites would find favourable conditions to become established and spread in the receiving human population.

## Authors' contributions

FG performed the statistical analysis and wrote the article. FS, HB and MB took part in the analyze of the data and the discussion about the results. SA did the immunological analysis. Dipsticks were designed by TE. FB, VF, LB, JFT PTK and MCH participated in the collection of data. MM and MD carried out the high-performance liquid chromatography. SB, TF, LA and BP participated in the discussion of the results. RL took part in the elaboration of the questionnaires. CR conceived the study, took part in the analyze of the data and the discussion and wrote the article. The final version of the manuscript was seen and approved by all authors.

## Supplementary Material

Additional File 1 Individuals and households' characteristics by site. Click here for file

Additional File 2 Sites characteristics. Click here for file

Additional File 3 Children's characteristics. Click here for file

Additional File 4 Chloroquine prevalence in urines of children between two and nine years of age according to site's characteristics. Click here for file

Additional File 5 Predicted and observed prevalence by site of the presence of chloroquine in children's urines. Click here for file
