# Supplementary material for: Antimalarial drug use in general populations of tropical Africa
Source: Malar J. 2008 Jul 8;7:124. doi: 10.1186/1475-2875-7-124 (PMC2494551; doi:10.1186/1475-2875-7-124)
Supplement: Additional File 5 — Predicted and observed prevalence by site of the presence of chloroquine in children's urines. [file 1475-2875-7-124-S5.doc]

Additional file 5: Predicted and observed prevalence of the presence of chloroquine in children’s urines. Logistic regression model taking into account the interdependency of observations made within the same site.

Numbers indicated at the bottom of the graph correspond to the different sites included in the study.
